# Post-Acute Sequelae of COVID-19 at 12 Months in Hospitalized Patients: A Colombian Cohort Study

**DOI:** 10.7759/cureus.106606

**Published:** 2026-04-07

**Authors:** Alvaro J Lora Mantilla, Laura A Parra-Gómez, Valentina Ortegón-Vargas, Mariam Posso Paz, Catalina Cáceres Ramírez, Alberto J Vasquez-Cadena, Maria C Ayala-Gutierrez, Maria C Amaya Muñoz, Maria P Blanco Rueda, Maria I Diaz Caraballo, Silvia J Villabona-Florez, Edgar D Gomez Laitton, Paul A Camacho Lopez

**Affiliations:** 1 Research Directorate, Fundación Oftalmológica de Santander (FOSCAL), Floridablanca, COL; 2 Medicine, Faculty of Health Sciences, Universidad Autónoma de Bucaramanga, Bucaramanga, COL; 3 Radiology, Universidad de Santander, Bucaramanga, COL; 4 Pathology, University of New Mexico, Albuquerque, USA; 5 Dermatology, Faculty of Health Sciences, Universidad Autónoma de Bucaramanga, Bucaramanga, COL; 6 Internal Medicine, Faculty of Health Sciences, Universidad Autónoma de Bucaramanga, Bucaramanga, COL; 7 Epidemiology, Faculty of Health Sciences, Universidad Autónoma de Bucaramanga, Bucaramanga, COL; 8 Internal Medicine, Fundación Oftalmológica de Santander (FOSCAL), Bucaramanga, COL; 9 Medicine, Faculty of Health Sciences, Universidad Autónoma de Bucaramanga, Floridablanca, COL

**Keywords:** cohort study, colombia, post-acute sequelae of covid-19, post-acute sequelae of sars-cov-2 infection (pasc), sars-cov-2 complications, vaccination

## Abstract

Background/objectives: Post-acute sequelae of SARS-CoV-2 infection (PASC) are a major source of long-term morbidity among COVID-19 survivors, particularly after hospitalization. However, information on PASC prevalence and associated factors at 12 months in Latin America remains limited. This study aimed to estimate the prevalence of PASC at 12 months among hospitalized COVID-19 patients and to identify factors associated with its persistence.

Methods: We conducted an ambidirectional, single-center cohort study including adult patients hospitalized with laboratory-confirmed SARS-CoV-2 infection who completed follow-up evaluations at six and 12 months after discharge. PASC was defined according to the World Health Organization (WHO) criteria. Sociodemographic variables, comorbidities, markers of in-hospital disease severity, and follow-up information were collected using standardized protocols. Statistical analyses included descriptive and bivariate comparisons according to PASC status at 12 months.

Results: Among 869 hospitalized COVID-19 survivors, the prevalence of PASC at 12 months was 40.7% (n=354). Female sex, markers of severe acute disease, and the presence of PASC at six months were significantly associated with PASC persistence at 12 months. Vaccination coverage at follow-up was high, and although adverse reactions to vaccination were more frequently reported among patients with PASC, no differences in PASC development were observed according to vaccine platform.

Conclusions: More than two out of five patients experienced PASC one year after hospitalization for COVID-19. Associations with severe acute disease and vaccine reactogenicity support the hypothesis that dysregulated host immune responses may contribute to persistent post-COVID symptoms, highlighting the need for early identification and longitudinal follow-up of high-risk patients.

## Introduction

The SARS-CoV-2 pandemic was one of the greatest health crises of the last century, not only because of its impact on mortality and health system collapse but also due to the substantial disability among survivors. Although the pandemic phase has ended, the disease burden persists. A considerable proportion of patients continue to experience persistent symptoms months after acute infection, constituting a multisystem syndrome known as long-COVID or post-acute sequelae of SARS-CoV-2 infection (PASC).

The World Health Organization (WHO) defines PASC as new, recurrent, or persistent symptoms occurring three months after probable or confirmed COVID-19, lasting at least two months, and not explained by an alternative diagnosis [1]. PASC has emerged as a new burden, with important clinical, functional, and socioeconomic repercussions, particularly among patients who require hospitalization for COVID-19 [2].

Reported prevalence of PASC varies widely, ranging from 10% to over 50%, depending on follow-up duration, operational definition, and severity of the initial infection [3]. In our previous work, 47% of patients had persistent symptoms at six months [4]. Symptoms were more frequent among women, patients with chronic obstructive pulmonary disease (COPD) or rheumatological diseases, and those who required hospitalization or mechanical ventilation during the acute phase [4]. These findings are consistent with international evidence identifying female sex, older age, higher body mass index, smoking, pre-existing comorbidities, and prior hospitalization or ICU admission as risk factors for PASC [5].

PASC is intrinsically heterogeneous, encompassing manifestations with variable and potentially overlapping etiologies, which may contribute to underdiagnosis. Clinical presentation differs across patients and can involve multiple organ systems, including respiratory, cardiovascular, and neurological domains [6]. This heterogeneity is compounded by the lack of a universally adopted definition and diverse diagnostic criteria across international health organizations (WHO, Centers for Disease Control and Prevention (CDC), National Institute for Health and Care Excellence (NICE)), which hinders uniform symptom identification [7]. Additionally, the absence of standardized diagnostic tools and validated biomarkers may lead to misdiagnosis or underestimation of prevalence, complicating clinical identification and subsequent management [8].

In Latin America, detailed studies assessing PASC prevalence at 12 months among previously hospitalized patients remain limited. Although studies from Brazil and Mexico have reported PASC prevalence from 37% to 77% in general populations, a comprehensive characterization of symptom persistence specifically at 12 months post-hospitalization is scarce [9,10]. This gap restricts understanding of the long-term trajectory of PASC and complicates appropriate care to affected patients, particularly in a region heavily afflicted by COVID-19. Therefore, the objective of this study was to estimate the prevalence of PASC symptoms at 12 months among patients hospitalized for COVID-19 and to identify variables associated with PASC [9-11].

## Materials and methods

Study design and setting

This study was part of the Fundación Oftalmológica de Santander (FOSCAL) COVID-19 cohort, an ambidirectional, single-center cohort established between March 2020 and September 2021 at FOSCAL, a tertiary-level referral hospital located in Floridablanca, Santander, Colombia (959 meters above sea level). The cohort was designed to collect clinical data prospectively from those with confirmed SARS-CoV-2 infection and to conduct longitudinal follow-up after discharge. 

The baseline characteristics, methodology, as well as in-hospital outcomes and mortality analyses have been previously published [4,12,13]. Additional analyses addressing hospital lethality and medium-term post-discharge outcomes have also been reported. 

Moreover, an earlier analysis of this cohort evaluating PASC at six months after hospital discharge has been previously reported [4]. The present study represents an extension of the cohort follow-up to 12 months, allowing a more comprehensive assessment of the persistence and evolution of symptoms over time.

Participants

All consecutive adult patients (≥18 years) admitted to FOSCAL with laboratory-confirmed SARS-CoV-2 infection by real-time polymerase chain reaction (RT-PCR) between 29 March 2020 and 27 September 2021 were eligible for inclusion. 

For the present analysis, we included patients who were discharged from in-hospital management for SARS-CoV-2 index infection and completed both six- and 12-month follow-up evaluations. Patients were excluded if they died before clinical assessment, had incomplete medical records for key variables, declined participation, or could not be contacted for follow-up.

Data collection and procedures

Data collection was conducted by trained research personnel using standardized electronic case report forms derived from institutional electronic medical records. LimeSurvey (LimeSurvey GmbH, Hamburg, Germany) was used to manage data collection, minimize missing entries, and facilitate real-time quality control. For patients discharged alive, structured telephone interviews and/or outpatient evaluations were conducted to complete follow-up data and to identify persistent or new symptoms after acute COVID-19. 

Initial evaluation included demographic information (age, sex, socioeconomic status), pre-existing comorbidities (coded via International Classification of Diseases, Tenth Revision (ICD-10)), acute COVID-19 clinical presentation, severity markers, hospital course, ICU admission and length of stay, and clinical outcomes, all extracted from electronic medical records [14]. The second and third evaluations involved telephone follow-ups at six and 12 months after the initial positive RT-PCR test, respectively, and included inquiry about persistent symptoms, new signs and symptoms, reinfection, new comorbidities, readmission, and rehabilitation therapies. 

Because the national COVID-19 vaccination campaign began after the index hospitalizations, vaccination in this cohort occurred during the post-discharge follow-up period; therefore, vaccination status was systematically collected only at the 12-month follow-up evaluation.

Definitions

PASC was defined according to the WHO definition and consistent with recent literature as the presence of persistent or newly developed symptoms occurring at least three months after the onset of SARS-CoV-2 infection, lasting for at least two months, and not explained by an alternative diagnosis [1].

Ethical considerations

The study protocol was approved by the Research Ethics Committee of FOSCAL (approval no. 03361/2020). All participants provided informed consent. The study was conducted in accordance with the Declaration of Helsinki and local regulatory requirements. 

Statistical analysis

Statistical analyses were performed using Stata version 19 (StataCorp LLC, College Station, USA). Before analysis, data quality was assessed by verifying the completeness and consistency of all variables of interest, with no missing data identified.

The distribution of quantitative variables was evaluated using the Shapiro-Wilk test, which indicated a non-normal distribution for all continuous variables; therefore, these variables were summarized using medians and interquartile ranges (IQRs), while categorical variables were described using absolute frequencies and percentages.

Bivariate analyses were conducted using the presence or absence of post-acute COVID-19 syndrome at 12 months as the dependent variable. Comparisons between groups were performed using the chi-square test for categorical variables, given that all expected cell counts were adequate, and the Mann-Whitney U test for quantitative variables. A two-sided p-value <0.05 was considered statistically significant.

## Results

**Figure 1:**
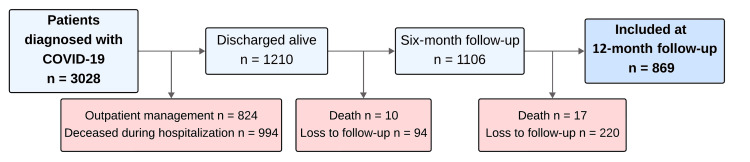
Flowchart of patient inclusion in the present analysis.

During the study period, 3,028 patients with SARS-CoV-2 infection were included in the overall cohort, of whom 2,024 required in-hospital management. A total of 1,210 patients were discharged alive, and 1,106 were contacted at six-month follow-up. At 12 months, 869 patients completed follow-up and were included in the present analysis (Figure 1). The prevalence of PASC at 12 months was 40.7% (n=354).

Table 1 summarizes patient characteristics stratified by PASC status at 12 months. The median age was 58 years (IQR 48, 67). Most participants had a medium-to-high socioeconomic status and secondary education level. Sex distribution was balanced overall; however, the proportion of female patients was significantly higher in the PASC group (54%, n=191 vs. 42.9%, n=221; p=0.001).

**Table 1:** Sociodemographic, clinical, in-hospital, and six-month follow-up variables grouped by the presence of PASC. CVD: cardiovascular disease (ischemic heart disease, heart failure, stroke, and peripheral arterial disease); COPD: chronic obstructive pulmonary disease; CKD: chronic kidney disease; IQR: interquartile range; ARDS: acute respiratory distress syndrome; PASC: post-acute sequelae of SARS-CoV-2 infection *Asthma, interstitial lung disease, and obstructive sleep apnea syndrome.

Variable	Total n = 869	No PASC (n = 515, 59.3%)	PASC (n = 354, 40.7%)	p-value
Female sex	412 (47.4%)	221 (42.9%)	191 (54%)	0.001
Age, median (IQR)	58 (48, 67)	59 (47, 68)	58 (49, 67)	0.99
Socioeconomic status				
Low	2743 (31.8%)	161 (31.6%)	112 (32.1%)	0.89
Medium-to-high	585 (68.2%)	348 (68.4%)	237 (67.9%)	
Education level				
None	21 (2.4%)	13 (2.5%)	8 (2.3%)	0.53
Primary	182 (21.1%)	100 (19.5%)	82 (23.4%)	
Secondary	386 (44.7%)	237 (46.3%)	149 (42.5%)	
University	274 (31.7%)	162 (31.6%)	112 (31.9%)	
Comorbidities				
CVD	75 (8.6%)	44 (8.5%)	31 (8.8%)	0.91
COPD	34 (3.9%)	13 (2.5%)	21 (5.9%)	0.011
Other pulmonary disease*	42 (4.8%)	21 (4.1%)	21 (5.9%)	0.21
Diabetes	170 (19.6%)	92 (17.9%)	78 (22%)	0.13
Obesity	255 (29.3%)	139 (27%)	116 (32.8%)	0.066
Dyslipidemia	162 (18.6%)	90 (17.5%)	72 (20.3%)	0.29
Hypertension	338 (38.9%)	199 (38.6%)	139 (39.3%)	0.85
Rheumatic disease	31 (3.6%)	17 (3.3%)	14 (4%)	0.61
CKD	38 (4.4%)	23 (4.5%)	15 (4.2%)	0.87
Charlson index ≥3	301 (34.6%)	186 (36.1%)	115 (32.5%)	0.27
Smoking	160 (18.4%)	97 (18.8%)	63 (17.8%)	0.70
Passive smoking	87 (10%)	51 (9.9%)	36 (10.2%)	0.90
Biomass exposure	106 (12.2%)	57 (11.1%)	49 (13.8%)	0.22
Alcohol consumption ≥1/week	140 (16.1%)	81 (15.7%)	59 (16.7%)	0.71
In-hospital management				
Days of hospital stay, median (IQR)	8 (5, 12)	8 (5, 12)	8 (5, 14)	0.096
ICU	180 (20.7%)	90 (17.5%)	90 (25.4%)	0.005
Days of ICU, median (IQR)	13 (6, 28)	12 (6, 23)	15 (7, 33)	0.97
Mechanical ventilation	100 (11.5%)	44 (8.5%)	56 (15.8%)	<0.001
Days of mechanical ventilation, median (IQR)	18 (10, 30)	16 (10, 31)	18 (9, 30)	0.97
Tracheostomy	50 (5.8%)	21 (4.1%)	29 (8.2%)	0.010
Pronation	180 (20.7%)	90 (17.5%)	90 (25.4%)	0.005
ARDS	196 (22.6%)	97 (18.8%)	99 (28%)	0.002
Shock	37 (4.3%)	16 (3.1%)	21 (5.9%)	0.043
Oxygen requirement at discharge	234 (26.9%)	127 (24.7%)	107 (30.2%)	0.069
Six-month follow-up				
PASC at six-month follow-up	458 (52.7%)	245 (47.6%)	213 (60.2%)	<0.001
Ambulatory care respiratory therapy	389 (44.8%)	246 (47.8%)	143 (40.4%)	0.032
Ambulatory care physical therapy	118 (13.6%)	69 (13.4%)	49 (13.8%)	0.85
Ambulatory care oxygen requirement	224 (25.8%)	132 (25.6%)	92 (26%)	0.91
Ambulatory care corticosteroid therapy	182 (20.9%)	114 (22.1%)	68 (19.2%)	0.30
12-month follow-up				
Vaccination	803 (92.4%)	472 (91.7%)	331 (93.5%)	0.31
AstraZeneca	81 (10.1%)	50 (10.6%)	31 (9.4%)	0.23
Pfizer	340 (42.3%)	188 (39.8%)	152 (45.9%)	
Janssen	75 (9.3%)	51 (10.8%)	24 (7.3%)	
Moderna	114 (14.2%)	72 (15.3%)	42 (12.7%)	
Sinovac	193 (24%)	111 (23.5%)	82 (24.8%)	
Complete primary vaccination scheme	783 (90.1%)	465 (90.3%)	318 (89.8%)	0.82
Vaccination adverse reaction	318 (39.6%)	165 (35%)	153 (46.2%)	0.001
Ambulatory care respiratory therapy between the six- and 12-month follow-up	333 (38.3%)	185 (35.9%)	148 (41.8%)	0.080
Ambulatory care physical therapy between the six- and 12-month follow-up	218 (25.1%)	114 (22.1%)	104 (29.4%)	0.016
Ambulatory care oxygen requirement between the six- and 12-month follow-up	146 (16.8%)	76 (14.8%)	70 (19.8%)	0.052
Ambulatory care corticosteroid therapy between the six- and 12-month follow-up	57 (6.6%)	27 (5.2%)	30 (8.6%)	0.057

At admission, the most frequent comorbidities were hypertension (38.9%, n=338), obesity (29.3%, n=255), diabetes (19.6%, n=170), and dyslipidemia (18.6%, n=162); however, none of these showed association with PASC at 12 months. Only COPD was more frequent in patients with PASC (5.9%, n=21 vs. 2.5%, n=13; p=0.011).

Regarding in-hospital severity, PASC was associated with severe acute disease. Patients with PASC more frequently required ICU admission (25.4%, n=90 vs. 17.5%, n=90; p=0.005), mechanical ventilation (15.8%, n=56 vs. 8.5%, n=44; p<0.001), and pronation (25.4%, n=90 vs. 17.5%, n=90; p=0.005). Furthermore, they developed higher rates of complications such as acute respiratory distress syndrome (ARDS) or shock.

At the six-month follow-up assessment, the presence of PASC was associated with PASC at 12 months (60.2%, n=213 vs. 47.6%, n=245; p<0.001); instead, a significantly lower proportion of patients with PASC received ambulatory respiratory therapy (40.4%, n=143 vs. 47.8%, n=246; p=0.032). Between six and 12 months, a higher proportion of patients with PASC received physical therapy (29.4%, n=104 vs. 22.1%, n=114; p=0.016), with no differences in other outpatient care requirements.

Vaccination status was available at the 12-month follow-up. About 90.1% (n=783) of patients had completed the primary vaccination scheme. Vaccine platform distribution was similar across groups; mRNA-based vaccines were the most frequently administered (42.3%, n=340). Notably, adverse reactions to vaccination were reported more frequently among patients with PASC compared with those without PASC (46.2%, n=153 vs. 35.0%, n=165; p=0.001); however, no significant differences were observed in the development of PASC according to vaccine platform.

**Figure 2:**
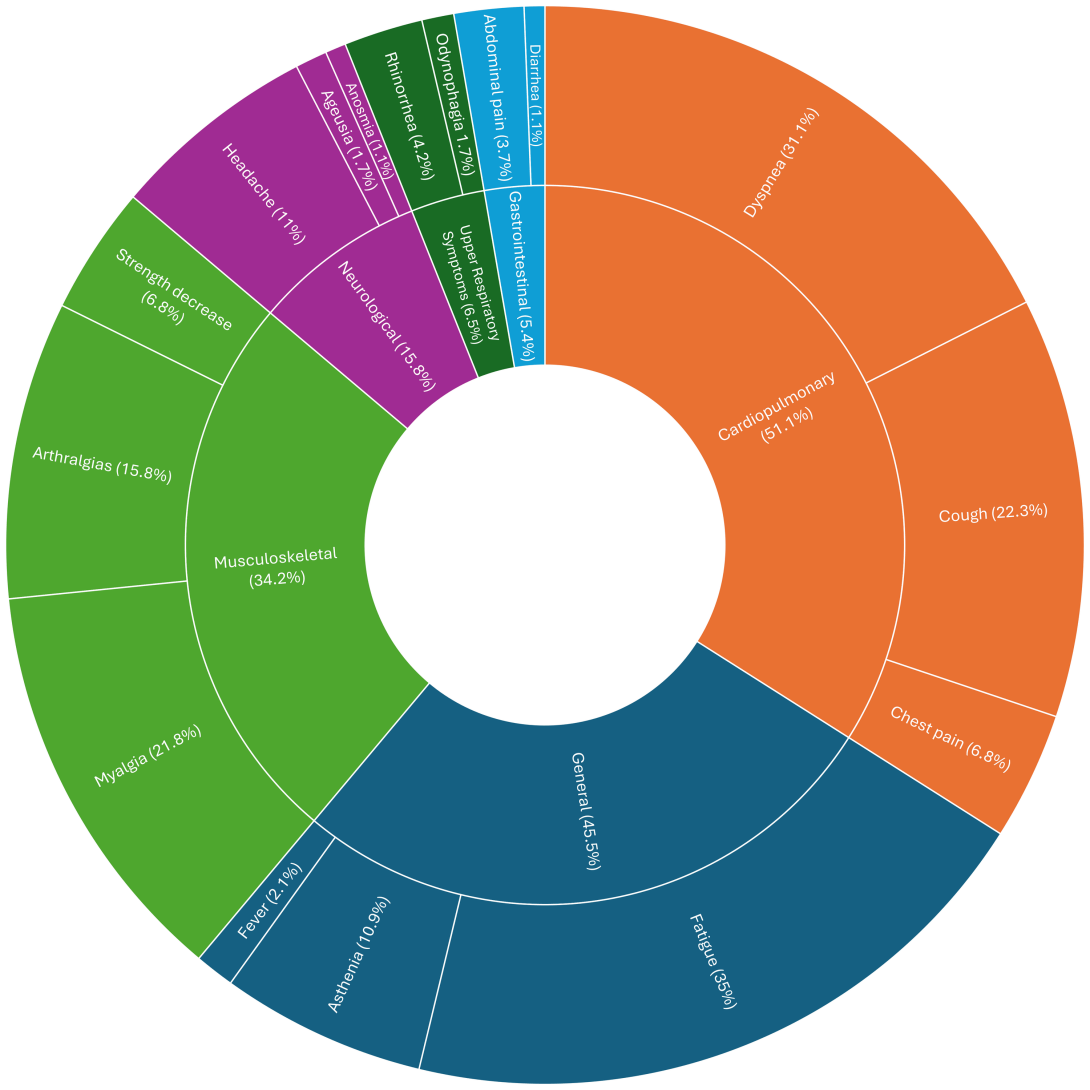
Sunburst chart showing symptom frequency in patients with PASC at 12-month follow-up. Some patients exhibited more than one symptom. PASC: post-acute sequelae of SARS-CoV-2 infection The figure was created in Microsoft Excel (Microsoft Corp., Redmond, USA) and refined using Inkscape (Inkscape Project, https://inkscape.org).

At 12 months, PASC presentation showed prominent multisystem involvement across six major domains (Figure 2). Cardiopulmonary symptoms were the most common (51.1%, n=181), with dyspnea as the leading symptom (31.1%, n=110), followed by persistent cough (22.3%, n=79) and chest pain (6.8%, n=24). General symptoms were reported by 161 (45.5%) patients; fatigue was the most common symptom overall (35.0%, n=135). Musculoskeletal symptoms occurred in 34.2% (n=121) of PASC patients, mainly myalgia (21.8%, n=77) and arthralgias (15.8%, n=56). These findings highlight a substantial burden of cardiopulmonary and constitutional symptoms one year after the acute phase, with symptoms overlapping across multiple organ systems.

## Discussion

We assessed PASC at 12 months among patients hospitalized with COVID-19 at our institution. Despite improvements in acute outcomes, long-term sequelae remained common, with a PASC prevalence of 40.7% (n=354). Female sex was significantly associated with PASC at 12 months (p<0.001), consistent with our prior six-month analysis, which included the full cohort (hospitalized and non-hospitalized patients) and identified female sex as a predictor of long COVID (prevalence 47.07%) [4].

However, this is in contrast with our baseline study, which evaluated acute disease across all cases and found male sex to be more strongly associated with hospitalization and severe illness [12]. Together, these findings suggest a shift from male predominance in acute severity to female predominance in long-term post-COVID symptoms, consistent with reports from diverse cohorts [15,16]. This may reflect sex-based differences in immune regulation and post-viral inflammatory responses [17].

Cardiopulmonary manifestations were the most prevalent symptom domain, with dyspnea as the leading symptom (31.1%, n=110), consistent with a large 12-month longitudinal cohort that followed both hospitalized and non-hospitalized COVID-19 patients in which shortness of breath remained a common persistent symptom [18].

Among hospitalized survivors (n=869), markers of acute severity were consistently associated with PASC at 12 months. Compared with patients without PASC, those with PASC more frequently required ICU care (25.4%, n=90 vs. 17.5%, n=90; p=0.005), invasive mechanical ventilation (15.8%, n=56 vs. 8.5%, n=44; p <0.001), developed ARDS (28.0%, n=99 vs. 18.8%, n=97; p=0.002), and developed shock (5.9%, n=21 vs. 3.1%, n=16; p=0.043). This aligns with high-quality international evidence showing that prior hospitalization/ICU admission and severe acute disease are key predictors of post-COVID condition/PASC [5]. It is also consistent with 12-month follow-up studies in critical COVID-19 survivors, which report substantial long-term morbidity after ICU-level disease and support the clinical plausibility that organ injury and critical illness trajectories (including ventilatory support and ARDS physiology) contribute to persistent symptoms well beyond discharge [19,20].

In contrast, diabetes (22.0%, n=78 vs. 17.9%, n=92; p=0.13), hypertension (39.3%, n=139 vs. 38.6%, n=199; p=0.85), and smoking (17.8%, n=63 vs. 18.8%, n=97; p=0.70) were not associated with PASC at 12 months. This is notable because our institution’s in-hospital lethality analysis identified hypertension, diabetes, and current smoking as risk factors for mortality during the index hospitalization (and thus strong markers of acute risk in this population) [13]. While evidence has suggested that smoking and pre-existing comorbidities may increase the risk of PCC in broader, mixed-severity populations [5], our results suggest that, in a cohort restricted to survivors of hospitalization, longer-term PASC risk may be more strongly discriminated by critical illness features than by traditional cardiometabolic risk factors. This may reflect mediation through acute severity, survivor selection, and/or differing PASC phenotypes across settings [21].

Our data support the hypothesis that the presence of PASC at six months may not reflect merely a “delay in recovery” but rather a phenotype prone to persistence, given its significant association with PASC at 12 months. This interpretation is consistent with longitudinal cohorts of hospitalized patients, in which improvement between approximately 5-6 months and one year is often partial, and a relevant subgroup continues to experience symptoms over time [9-11,18-20]. Taken together, these findings suggest that some survivors may enter a more stable or slowly resolving post-acute phase, so identifying PASC as early as six months may be useful for risk stratification and follow-up planning [22].

In addition, we found that participants who reported adverse reactions to COVID-19 vaccination had a higher risk of PASC at 12 months. This is biologically plausible, as vaccine reactogenicity reflects immune activation and inflammatory signaling following antigen exposure. Although there is still limited direct evidence to support reactogenicity as a predictor of long COVID, our finding is consistent with the hypothesis that PASC involves persistent immune perturbations. In this context, greater reactogenicity could serve as a clinical indicator of an immune system more prone to exaggerated or prolonged responses, a possibility that should be confirmed in prospective studies using standardized reactogenicity measures and detailed immunophenotyping [23-27].

Our study has several methodological limitations that should be acknowledged. First, the nature of the studied outcome (PASC) lacks objective and fully reproducible diagnostic criteria. Although follow-up assessments were conducted by trained research personnel familiar with standardized definitions of the condition, the etiology of certain reported symptoms may have been attributable to other conditions that were not evaluated or diagnosed by the healthcare team. Second, baseline and hospitalization data were retrospectively extracted from medical records; therefore, some relevant information may not have been captured or may have contained documentation or entry errors. In addition, follow-up data were based on self-reported information, which is inherently susceptible to recall bias; nevertheless, given the subjective and heterogeneous nature of PASC manifestations, self-reporting remains the most feasible approach for outcome ascertainment in this context. Finally, the cohort was designed and implemented during the early phase of the COVID-19 pandemic, which resulted in the omission of certain variables that later became clinically relevant, such as vaccination status at baseline; because national vaccination campaigns began in late March 2021, when several six-month follow-up interviews had already been completed, vaccination data were systematically collected only at the 12-month follow-up to preserve data homogeneity across participants.

Nevertheless, our study has many strengths that add value to the presented findings. First, the cohort was implemented within the context of the COVID-19 pandemic, allowing for real-time inclusion of patients with confirmed SARS-CoV-2 infection by positive PCR testing, and follow-up assessments were systematically conducted by trained research personnel using a strictly structured protocol, including predefined contact windows and data auditing procedures to ensure data quality. In addition, the longitudinal design with standardized follow-up at six and 12 months enabled the evaluation of symptom persistence and evolution over time. Second, this cohort represents one of the largest reported in Colombia and Latin America, both at hospital admission and during follow-up. Moreover, the study was conducted in a middle-income country at a tertiary referral center in northeastern Colombia that serves a broad and heterogeneous population, providing high-quality evidence from a region that remains underrepresented in the PASC literature, thereby enhancing the external validity and generalizability of the results.

## Conclusions

In this cohort of hospitalized COVID-19 survivors from a middle-income country, PASC remained highly prevalent 12 months after acute infection, affecting more than two out of five patients. Female sex and markers of severe acute disease were consistently associated with long-term PASC, highlighting the role of acute illness severity in shaping prolonged recovery trajectories. Importantly, the presence of PASC at six months was strongly associated with persistence at 12 months, suggesting that early post-acute symptoms may identify a subgroup at risk for sustained morbidity. Although adverse reactions to COVID-19 vaccination were more frequently reported among patients with PASC, no differences in PASC development were observed according to vaccine platform. Taken together, the higher frequency of PASC among patients with severe acute infection and those reporting vaccine reactogenicity supports the hypothesis that an exaggerated or dysregulated host immune response may contribute to the pathophysiology of persistent post-COVID symptoms. These findings emphasize the substantial long-term burden of PASC among hospitalized patients and support the need for structured follow-up strategies and targeted rehabilitation programs, particularly in regions that remain underrepresented in the global PASC literature.
